# Investigation of the Durability of Gaskets in Contact with Bio- and Aviation Fuels

**DOI:** 10.3390/ma15186288

**Published:** 2022-09-09

**Authors:** Grzegorz Romanik, Janusz Rogula, Paweł Regucki

**Affiliations:** Department of Energy Conversion Engineering, Faculty of Mechanical and Power Engineering, Wrocław University of Science and Technology, Wybrzeże Wyspiańskiego 27, 50-370 Wroclaw, Poland

**Keywords:** gasket, tightness, leakage, biofuel, aviation

## Abstract

Care for the natural environment, which can be observed in the tightening of emission standards, has enforced the search for new fuels, especially renewable sources of natural origin. The article presents the results of theoretical and experimental considerations on the impact of aviation biofuels on the materials used for sealing flange joints. The fuel type selected for the test is compatible with aviation fuels. Fuels have been enriched with a bio-additive that changes the technical and physical properties of the fuel. The tested gaskets were made of soft, aramid-elastomeric materials that were flat in shape and without reinforcement. Their commercial names are AFO and AFM. Tests were carried out with the use of a simple flange joint with a fuel reservoir at 373 K. Both fuel loss and the pressure drop on the gasket were measured during a 1000 h period of time. The experiments showed that the seals preserved the technical parameters in the presence of the tested fuels. The fuel loss did not exceed the accepted limits, which demonstrates the suitability of the tested materials for utilization with new types of fuel. However, no unequivocal conclusions can be drawn about the positive or negative impact of bio-additives on the sealing material due to the fact that both an improvement and deterioration in tightness under certain circumstances were observed. Based on the experimental data, a mathematical model was proposed that makes it possible to predict the service life of the gaskets in flange joints in contact with the investigated types of fuel. The potential application of the research results is practical information about the impact of biofuel on the gasket, and hence the information about the possibility of using traditional sealing materials in a new application—for sealing installations for the production, transmission and storage of biofuels.

## 1. Introduction

Biofuels are considered as an alternative source of energy. For economic and ecological reasons, biofuels are increasingly used for combustion engines [1]. The application (in the automotive industry) of biofuels made of waste animal fat was considered based on laboratory scale experiments on combustion engines, as well as on full scale tests from vehicle fleets. Previous research has shown an engine power loss of 8% [2,3,4,5] with a slight increase in fuel consumption [6,7,8]. However, it is possible to decrease fuel consumption and improve the composition of exhaust gases by modifying the unit settings of electronic controls [9].

Aviation is one of the highest sources of pollutants in the transport sector. The demand for air transport services is constantly increasing, and the demand for jet fuel in the world is predicted to increase by about 38% during the period of 2008 to 2025 [10]. At the same time, according to the United Nations Framework Convention on Climate Change, the airline sector is currently responsible for 3% of the total global greenhouse gas emissions. These are the reasons why new alternative aviation fuels are investigated with regard to replacing conventional fuel. Among others, hydrogen fuel, liquefied fuels (e.g., propane, butane), alcohols (e.g., ethanol, methanol), biofuels (combustible liquid manufactured from renewable sources, such as animal fats and plants oils) and synthetic fuels (fuel produced from the synthesis process, such as the Fischer–Tropsch process) are considered [11]. An interesting option is to replace bioethanol with biobutanol in the blend, as it has numerous advantages, e.g., it is 25% more caloric when compared to bioethanol; it is less hygroscopic, less corrosive, and less aggressive to fuel systems; and most importantly, it emits less pollutants when burned [12,13]. The sustainability of the crops used to produce biofuel is important to ensure that the production of feedstock does not interfere with the supply of food or freshwater, and in turn contribute to higher food prices due to competition with food crops. Other research has focused on ensuring that biofuels will not cause any anthropogenic problems due to deforestation during the creation of sufficient farm land. With regard to the properties of biofuels, their calorific value, combustion stability and freeze point should be carefully assessed.

The aviation industry has been attempting to improve the operation of engines. However, airline emissions are expected to grow at a rate faster than the industry can improve fuel efficiency. Research has reported that the benefits of using biofuels in aircraft engines are measurable and that no detrimental effects on the engine’s mechanical components are noticed, even if the content of the bio-additive in the fuel is several dozen percent [10,11,14]. The emission of gaseous pollutants such as CO, CO_2_ and NOx from a turbine engine can be reduced by an average of 30–70% when replacing fossil fuels with biofuels [15,16]. Biofuel is a suitable choice that could not only significantly lower GHG emissions, but which is also a renewable source of energy. Moreover, the price of fossil fuels is growing rapidly, and therefore biofuels can be a way to reduce transport costs.

The origin of biofuels can be vegetable or animal. The ecological aspects of biofuel production were discussed in [17]. Human activity generates over 2 billion tons of solid and liquid organic wastes, and therefore recycling processes with aspects of producing or recovering gaseous energy have become crucial on a global scale. There is a need for a deep and multi-directional analysis of the problem in order to avoid the net profit for the environment deteriorating from the utilization of biofuels [18,19]. The application of new fuel requires a wide analysis of the impact on the fuel system of an engine and its combustion chamber.

In the case of a fuel supply system, elastomer seals are commonly used. In older constructions, nitrile rubbers have been successfully used. However, contact with bio-additives has been found to accelerate the degradation of the elastomer, e.g., swelling, hardening, and relaxation [20,21,22,23,24,25,26]. It refers to the rubber seals. Another type of seal that was tested in the environment of biofuel was cork rubber flat gasket [27]. In the fuel industry, in refineries, in production and transmission installations, flange joints of pipelines are sealed with flat gaskets which are composites of fiberglass, mineral, aramid, fillers, nanofillers, connected with a NBR rubber binder. Therefore, the use of a different fuel requires the analysis of the chemical compatibility of the sealing material and, if necessary, some modifications should be implemented. Failure to do so may lead to leakages, and thus to the risk of fire and environmental contamination. Another no less important factor is the transport of the fuel. The transport system also needs to be compatible with the new bio-fuel. Fuel must be transported over both short or longer distances by pipelines, or cisterns that are stored in sealed tanks. In such installations, flange joints are a common connecting element. In industrial installations, flange joints are responsible for over 5% of the total leakage emission [28]. It is assumed that there are about 100,000 such connections in a medium-sized refinery.

A demountable joint can be seen to be a potential source of leakage, and its leakage level should be determined at the design stage [29]. In the design phase, an appropriate tightness of the flange joint must be achieved by a combination of force in the bolts (achieved by the tightening torque, which is connected with the friction in the threads, the bolt head and on the strip), the stiffness of the flange, and the working range of the tightness of the selected gasket (each gasket has a minimum and maximum pressure—under minimum pressure, undesired leakage may occur, and after maximum pressure the seal may be destroyed). Problematic cases can occur, for example, when the flange joint is designed at the limit of the stiffness of the flange, and at the same time at the lower limit of the seal’s tightness. In these cases, it is very difficult to achieve the tightness of the flange connection during assembly [30]. The transporting medium in most cases affects living organisms, so even fugitive emission should not be neglected [31,32]. The durability of a flange joint is connected with the type of load it experiences. It is quite disadvantageous to subject the flange joint to a vibration load, with the joint’s durability being considerably shorter when compared to being subjected to a static load [33]. Moreover, the gasket used in a flange joint is subjected to the same physical phenomena, regardless of its design [34,35,36,37].

The importance of the durability tests of seals in contact with biofuels results from the specific properties of biofuels. The production and transport of the fuels requires a system of pipelines and valves, where flange gaskets are commonly used. Due to the specific parameters of biofuel, the pipeline system and gaskets are exposed to [1]:

- A reduced durability of the elements that are in contact with the fuel, which are made of typical elastomers and rubbers;
- The corrosion of paint coatings on elements in contact with the fuel;
- A strong corrosive effect on copper-containing alloys;
- Contact with water (e.g., bioethanol is hygroscopic and can absorb water from both the distribution system and the surrounding air, in turn increasing the corrosion tendency of the steel and metal construction materials in the fuel system);
- Sludge and precipitation;
- Microbiological contamination.

Considering all the above mentioned arguments, the authors strongly believe that further investigations of the seal material that is in constant contact with biofuels are fully justified due to the different properties of biofuel when compared to traditional fuels.

## 2. Materials and Methods

### 2.1. Test Stand

**Figure 1:**
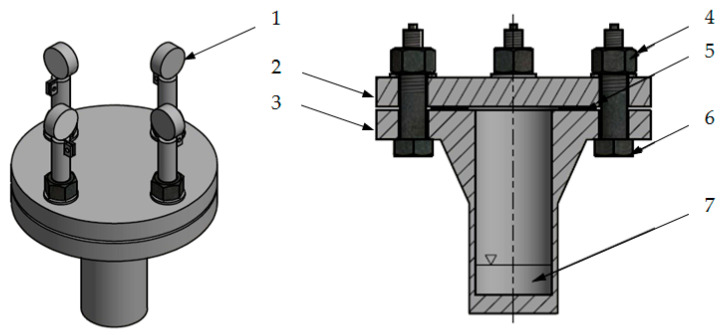
Scheme of the test stand: 1—dial gauge, 2—upper flange, 3—lower flange, 4—nut, 5—gasket, 6—bolt, 7—20 mL chamber with fuel.

The test stand was constructed on the basis of a DN40 flange. Figure 1 presents the schematic view of the stand.

The stand consists of two flanges: the upper (2) and the lower (3), between which a flat gasket (5) is mounted—the tested object. The flanges are pressed together with four bolts (6). The appropriate tightening torque of the bolts ensures the required pressure on the gasket. The bolts have already been calibrated, thanks to which their force-elongation characteristics are known—equal to 0.443 kN per 1 µm of elongation. Dial gauges enable the elongation to be controlled, and also the tension of the bolts. In this way, the pressure on the gasket is controlled. There is a small fuel chamber (7) in the lower part. Weight loss indicates leakage from the flange joint, which is only possible in one way—through the gasket.

### 2.2. Tested Gaskets

**Figure 2:**
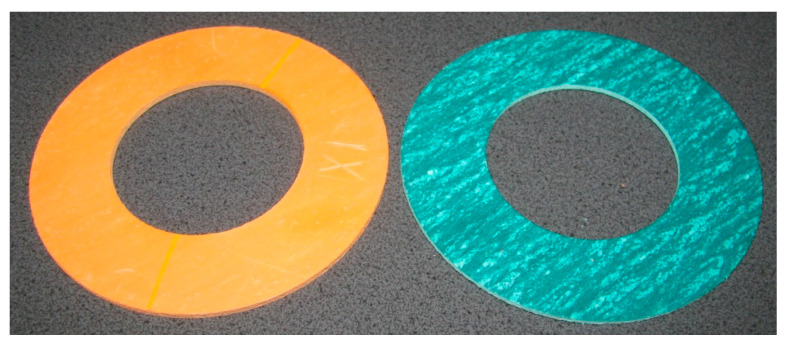
View of the tested gaskets; AFM—on the left, AFO—on the right.

Two types of gaskets were selected for laboratory testing: AFO and AFM. The gaskets were flat in shape, soft, aramid-elastomeric, and without reinforcement (Figure 2). The diameters of the gaskets were ø90 × ø50 × 2 [38].

The AFO material is a multi-layer composite, with aramid and mineral fibers that improve strength and thermal resistance. NBR rubber plays the role of the binder, and fillers ensure tightness. The AFM material is a composite with aramid fibers with nanofillers and HNBR rubber. The materials of the mentioned gaskets are suitable for classic fuels.

### 2.3. Tested Fuels

The following fuels were used in the research:

- Aviation fuel Jet A-1 (hereinafter referred to as Jet A-1),
- Aviation fuel Jet A-1 with 7.0% of fatty acid methyl esters (FAME) additive (hereinafter referred to as Bio-Jet A-1),
- Aviation gasoline Avgas 100 LL (hereinafter referred to as Avgas),
- Aviation gasoline with 4.9% of ethyl alcohol additive (hereinafter referred to as Bio-Avgas).

### 2.4. Testing Procedure

The laboratory tests were carried out based on the developed procedure in accordance with DIN 28090-3: 2014-11 [39].

**Figure 3:**
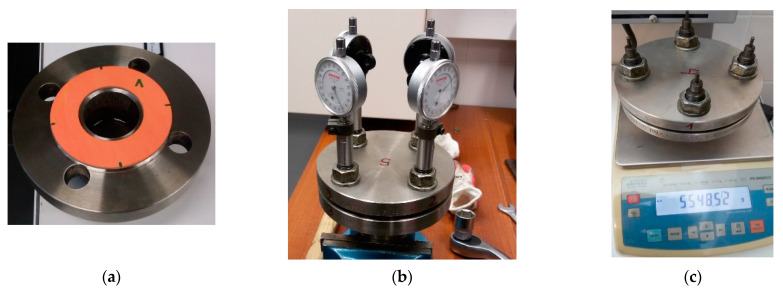
Research stages: (**a**) gasket assembly, (**b**) tension control of bolts, (**c**) weight control.

The geometric and mass features of the test objects were determined and then placed in the test flange joint, which served as a 20 mL vessel filled with the biofuel (Figure 3a). The initial mass of fuel was equal to 16.1 g in case of Jet A-1 and 14.4 g in the case of Avgas fuel. Then, after applying the second flange, the flange joint was tightened with marked bolts in order to obtain a sealing pressure of 30 MPa (Figure 3b). After removing the clock indicators, the mass of the assembled flange joint was determined (Figure 3c) and then inserted into the heating chamber at a temperature of 373 K. The heating time was 1000 h. At intervals of 24, 100, 200, 500 and 1000 h, the flanges were removed from the chamber in order to determine the weight loss of fuel over time, with the bolt tension then being checked to determine the actual pressure on the gasket [1].

The bolts used in the tests were calibrated. In order to elongate the bolts, they were tightened. The determined constant of the bolt elongation was 0.443 kN/µm. By knowing the bolt elongation, it is possible to determine the force *F*. Contact pressure *p* is described by the following formula:

(1) $$p=\frac{F}{S},$$

where: *F*—force in bolts, [N]; and *S*—gasket area, [mm^2^].

### 2.5. Leakage Determination

The mean diameter of the gasket can be calculated with the following formula:

∅_mean_ = (∅_outer_ + ∅_inner_)/2 (2)

The mean gasket’s circumference *L* is:

*L* = ∅_mean_⋅π (3)

The leakage rate *λ* is calculated from formula:

(4) $$\lambda =\frac{m}{L\cdot t},$$

where: *m*—biofuel mass loss, [mg]; *L*—mean gasket’s circumference, [m]; *t*—test time, [3,600,000 s].

### 2.6. Contact Pressure Model

As the presented research results are a continuation of research on the influence of the biofuel environment on fiber-elastomeric gaskets, it was decided to verify the previously created mathematical model. Only key fragments of the issue are presented [1].

The analytical descriptions of the rheological process occurring in a flange bolted joint gasketed with a soft material are very generic due to several effects:

- The creep process in a particular joint’s elements (flanges, bolts) proceed at a variable stress,
- The stress relaxation in a joint’s elements decrease at a changeable strain,
- A gasket exhibits the nonlinear dependence between stress–strain,
- The anisotropic properties of the gasket’s material,
- A higher differentiation of elastic modulus of the gaskets when compared to the flanges, bolts and nuts.

**Figure 4:**
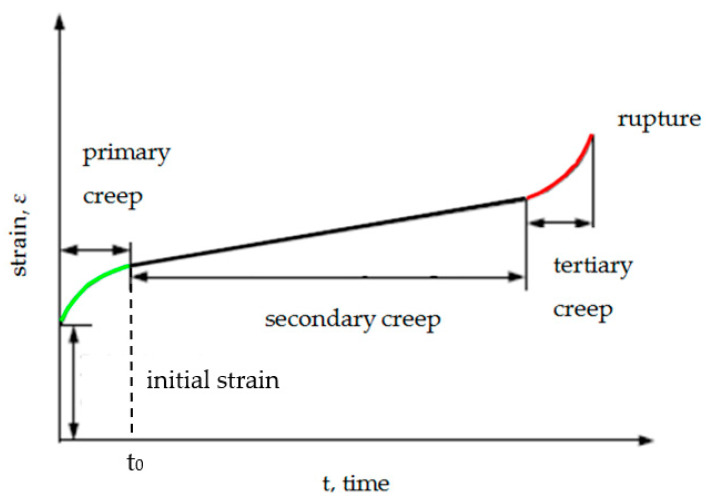
The creep process in the function of time [9].

In this paper, the viscoelastic properties of a gasket operated in flange bolted joints is described with regard to the power law. Based on this law, the strain’s intensity was categorized in three levels in accordance with Figure 4 [9].

The first stage of the process is so called preliminary creep, which occurs with a very high rate and in a short period of the time (highly non-linear process). The secondary creep occurs when the strain increases with a constant rate. The third field is called tertiary creep, and occurs when the strain rate proceeds in an uncontrolled way that finally causes the failure of the elements.

The strain in the second stage can be described by the following formula, where constants *B* and *n* are determined experimentally.

(5) $${\dot{\varepsilon }}_{creep}=B\sigma^{n}$$

The typical value of the *n* constant is bigger than 1 and usually does not exceed the value of 8. The higher the *n* value, the smaller the creep effect.

In order to simulate the gasket’s contact pressure (stress relaxation), the authors considered the following assumptions in relation to the tested flange joints:

- The bolts, nuts and flanges are very stiff, and the effect of their relaxation can be negligible,
- The gasket is a linear material with a constant elasticity modulus value,
- The strain rate occurs only in the gasket material, which in turn causes a decrease in the contact pressure.

Based on the above assumptions, the total strain of the gasket material can be written as:

(6) $$\varepsilon_{tot}=\varepsilon_{elast.}+\varepsilon_{creep}$$

Assuming that the gasket’s stress relaxation occurs at a constant strain, after derivation, Equation (5) can be written as:

(7) $$0={\dot{\varepsilon }}_{elast.}+{\dot{\varepsilon }}_{creep}$$

By substituting in Formula (6) the elastic strain considered as:

(8) $${\dot{\varepsilon }}_{elast.}=\frac{\dot{\sigma }}{E}$$

and by taking into account Equation (4), the contact pressure *σ*, which is dependent on time, can be written as:

(9) $$0=\frac{\dot{\sigma }}{E}+B\sigma^{n}=\frac{1}{E}\frac{d\sigma }{dt}+B\sigma^{n}$$

### 2.7. Characteristics Fitting

The experimental data allow the fitting of the theoretical curve that describes the changes of contact pressure *σ* with time—described by Equation (9). The shape of the theoretical characteristic reflects the rapid decrease of contact pressure during the first 200 h, and stabilization of its value latter on. The curve also predicts the asymptotic behavior of the observed contact pressure, which is confirmed by the experiments. The characteristic fitting is described by formula:

(10) $$\sigma \left(t\right)=\frac{t}{A\cdot t+B}+C$$

where constants {*A*, *B*, *C*} are the experimental parameters reflecting the individual features of the gasket’s material and the mutual interaction of this material with the fuel.

The physical interpretation of the *C* parameter is simple, because for *t* = 0 (initial state), this value refers to the initial contact pressure. Parameter *B* describes the rapid decrease of contact pressure during the preliminary creep stage. Later on, when the time is tending to infinity (~1000 h), *B* becomes irrelevant. Moreover, parameter *A* has a large influence on the asymptotic value of the contact pressure:

(11) $$\sigma_{asym.}=\underset{t\to \infty }{\lim}\frac{t}{A\cdot t+B}+C=\frac{1}{A}+C$$

All the parameters {*A*, *B*, *C*} are calculated from experimental data based on the least square approximation method. The characteristics are calculated for the pure Avgas and Jet A-1 fuels and then compared with the experimental results obtained for these fuels with bioadditives.

Based on the theoretical characteristic of the contact pressure, it is possible to estimate the time *t*_0_ for when the secondary creep starts. The secondary creep is characterized by the linear increase of the strain *ε*, which is marked by the black line in Figure 4. This stage is reflected in the linear decrease of the contact pressure at the characteristic described by (10). By fitting the straight line *y*(*t*) to the last part of the characteristic (e.g., *t* ∈ (600, 1000)) using the least square approximation method, it is possible to calculate where this line begins to deviate from the tested characteristic by calculating the relative error:

(12) $$\epsilon =\left|\frac{\sigma \left(t\right)-y\left(t\right)}{\sigma \left(t\right)}\right|\cdot 100\%$$

The time *t*_0_ could be estimated by assuming the limit value of ϵ=2%.

## 3. Results and Discussion

### 3.1. Contact Pressure

During the interval times after 24, 100, 200, 500 and 1000 h of the testing, dial indicators were screwed onto calibrated bolts and then zeroed. After quickly unscrewing the nut and archiving the bolt elongation value, the screw was reassembled so that the probe indicator clock showed zero again. These measurements allowed the contact pressure on the gaskets to be determined. The results of the contact pressure drop of the AFO and AFM gaskets are presented in Table 1 and Table 2.

There was a noticeable drop of contact pressure on all the gaskets during the first 24 h. In the case of the AFO material, it reached the level of 18.5 MPa for the Bio-Avgas and 18.2 MPa for the Avgas, respectively. For the same period of time, in the case of the Jet A-1, the contact pressure was at the level of 17.8 MPa in the presence of bioadditives, and 17.4 MPa without additives. This means that bioadditives at the first stage (primary creep) preserve contact pressure even better than pure aviation fuels.

The AFM material was characterized by the following values of contact pressure: 19.5 MPa for the Bio-Avgas, and 16.4 MPa for the Avgas without bioadditives. This confirms the observations made for the AFO material. However, in the case of the Jet A-1, the opposite behavior was reported. The contact pressures were equal to 16.6 MPa for the Bio-Jet A-1, and 18.7 MPa for the pure Jet A-1.

After 24 h, the contact pressure’s drop was not so rapid, which means that the creep phenomenon became smaller. In the period from 24 to 200 h of the tests, the creep gradually decreased, in turn preserving the much smaller character of the drop. The reduction in contact pressure in the case of the AFO gasket was at the level of 23.4% for the Bio-Avgas, and 24.2% for the Avgas without bioadditives. For the Jet A-1 fuel, it was 25.3% and 20.1% with the bioadditives and without the bioadditives, respectively. By analyzing the above data, it can be seen that the bioadditives in the Jet A-1 fuel deteriorated the properties of this fuel when in contact with the AFO gasket. In turn, the bioadditives in the Avgas fuel improved the properties of this fuel when compared to the pure Avgas fuel. These data indicate that the Bio-Avgas is more predisposed for the AFO gasket than the Bio-Jet A-1.

In the case of the AFM material, for the analyzed 24–200 h period of time, the following values were obtained:

In the case of the Avgas, there was a 15.9% and 22.6% contact pressure drop with and without the bioadditives, respectively.

In the case of the Jet A-1, the reduction of contact pressure was 18.7% and 26.7% with and without the bioadditives, respectively.

When analyzing the above data, it is clear that the bioadditives, when added to the pure fuels, improved their qualities. These data indicate that the Bio-Avgas is more predisposed for AFM material than the Bio-Jet A-1–as is the case with the AFO material.

The last investigated interval (500–1000 h) shows the long-term interaction of the analyzed fuels with the gasket materials. It is interesting that for the AFO material and the pure fuels (Jet A-1, Avgas), the contact pressure only dropped by about 3.1–4.1%, while for the same fuels with the bioadditives the decrease was 34.3 and 22.3% for the Jet A-1 and Avgas, respectively. For the AFM material, the long-term interaction with the pure fuels caused a contact pressure drop of 13.7 and 41.7% for the Jet A-1 and Avgas, respectively. The bioadditives had an ambiguous effect and caused the contact pressure drop of 22.7 and 21.2% for the Jet A-1 and Avgas, respectively. The changes discussed above are summarized in Table 3.

The values in Table 3 indicate that the AFO gasket was the best at preserving its properties (in the long term) in the presence of the pure Avgas and Jet A-1 fuels. In the case of the bioadditives, the largest contact pressure drop occurred for the Bio-Jet A-1, which means that this biofuel should not be used with the AFO gasket. The opposite situation occurred for the Avgas. For the pure Avgas, a large deterioration of the contact pressure was noticed when in contact with the AFM material (41.7%), while in the case of the AFO material, the contact pressure loss was negligible (4.1%). For this fuel, the bioadditives have an opposite effect on the long-term behavior of the AFO and AFM gaskets. Generally speaking, in the presence of the bioadditives, in the case of the AFO material, a significant deterioration of contact pressure was noticed, while for the AFM material, an improvement of contact pressure was observed.

**Figure 5:**
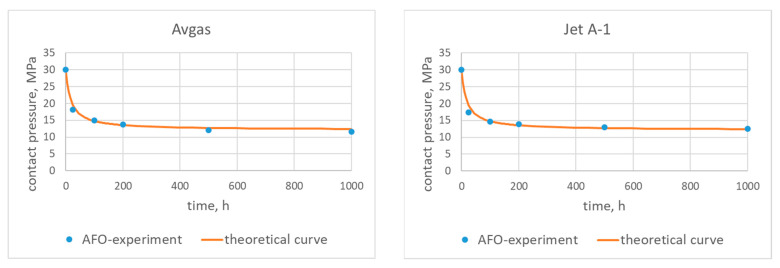

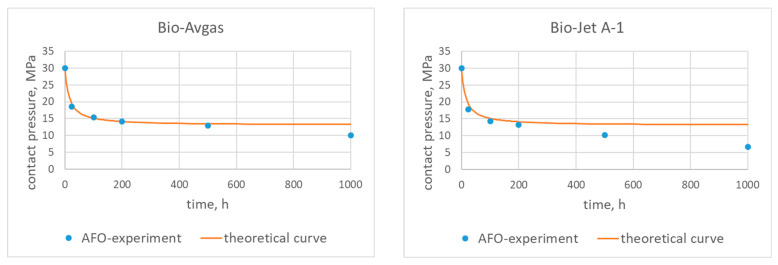
Contact pressure for the AFO gasket and the tested fuels—theoretical characteristics and experimental data.

**Figure 6:**
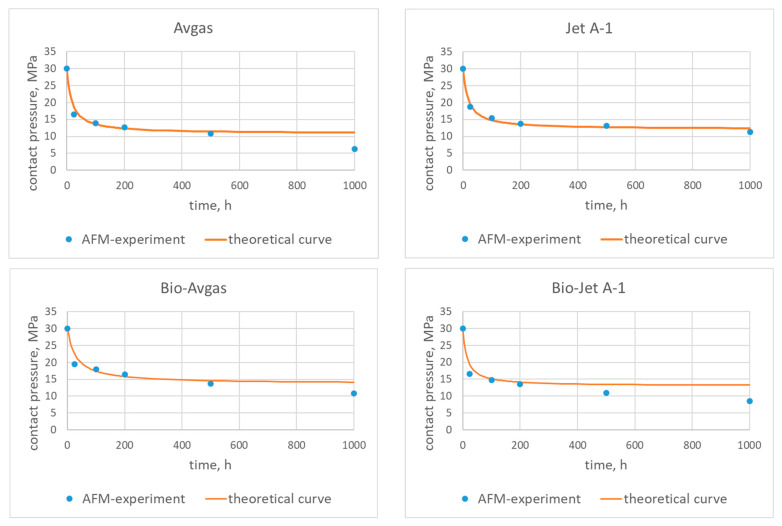
Contact pressure for the AFM gasket and the tested fuels—theoretical characteristics and experimental data.

A comparison of the experimental data with the theoretical characteristics calculated from (10) is presented in Figure 5 and Figure 6.

The coefficients {*A*, *B*, *C*} presented in Table 4 were fitted to the experimental data using the least square approximation method.

When analyzing the characteristics for the AFO and AFM materials fitted for the pure fuels, it is easy to see that both curves fit perfectly to the experimental data for the AFO gasket. It is also important to underline that the largest differences between the characteristics and the experimental data were for the pure Avgas in contact with the AFM gasket. This may indicate that the used AFM material is not suitable for this type of fuel.

The theoretical characteristics for the fuels with the bioadditives presented in Figure 5 and Figure 6 show the long term influence of the modified fuels on the AFO and AFM materials (*t* > 500 h). For the Bio-Jet A-1, a deterioration of contact pressure was observed for both types of gaskets. In the case of the Bio-Avgas, the changes were not so obvious. The modified Avgas better preserves the contact pressure with the AFM material than its pure version. On the other hand, however, a deterioration of contact pressure was observed for the AFO gasket (as was the case with the Bio-Jet A-1).

**Figure 7:**
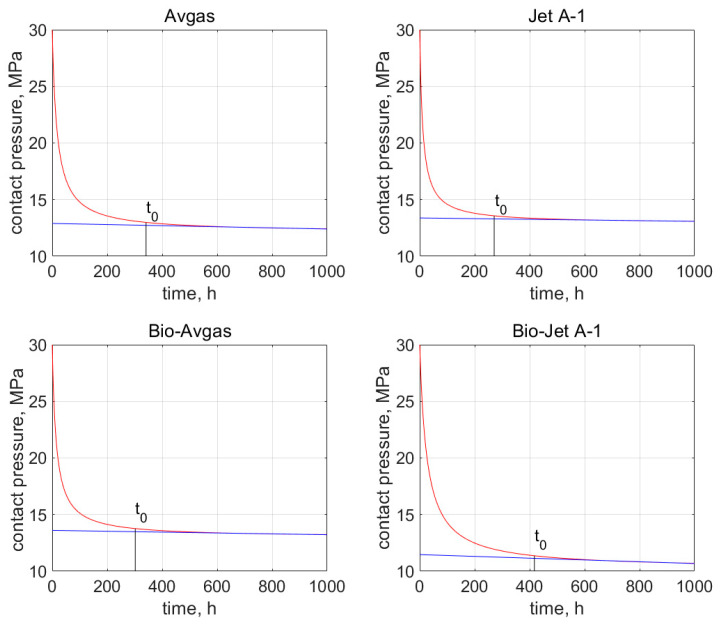
Time *t*_0_ from when the secondary creep starts, which is marked in the contact pressure graph (red curve) for the AFO gasket and all the tested fuels. The blue line indicates the linear decrease of constant pressure.

**Figure 8:**
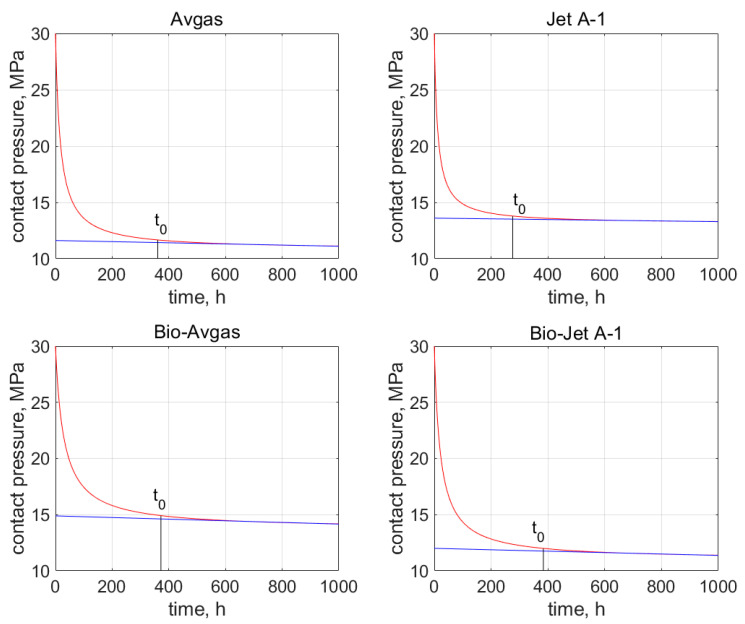
Time *t*_0_ from when the secondary creep starts, which is marked on the contact pressure graph (red curve) for the AFM gasket and all the tested fuels. The blue line indicates the linear decrease of constant pressure.

Moreover, the first part of these curves (i.e., *t* < 400 h) accurately reproduces the primary creep phenomenon presented in Figure 4. These characteristics also allow for the estimation of time *t*_0_, after which the contact pressure stabilizes at around a constant value, which in turn indicates the secondary creep stage. The values of *t*_0_ are determined on the basis of the relative error, which is described by Equation (12) and marked on the graphs shown in Figure 7 and Figure 8.

For the AFO gasket, the time *t*_0_ was 341 h for the Avgas, and 271 h for the Jet A-1. For the bioadditives, it was 302 and 417 h for the Avgas and Jet A-1, respectively. For the AFM gasket, the time *t*_0_ was 361 h for the Avgas and 276 h for the Jet A-1, while for the bioadditives it was 372 and 384 h for the Avgas and Jet A-1, respectively. In the case of the pure fuels, the value of time *t*_0_ was rather stable and fixed for both the AFO and AFM material. The bioadditives in the Avgas shorten the *t*_0_ in the case of the AFO gasket, or have no effect on this value in the case of the AFM. The opposite situation was observed for the Bio-Jet A-1. Moreover, a significant shift of *t*_0_ towards a value of around 350 h was observed for both materials. This effect is associated with a significant reduction in the contact pressure value.

### 3.2. Fuel Leakage Rate

The leakage rate *λ* was determined by taking into consideration the loss of fuel weight during the test. The results of the change in the mass of the biofuel during the tests are presented in Table 5 and Table 6.

The dimensions of the AFO gasket were as follows: its outer was equal to 90 mm, and its inner was equal to 50 mm. Since the presented research is a continuation of the research described in [1], the following calculations can be quoted. The mean diameter of the gasket can be calculated with the following Formula (2):

∅_mean_ = (90 mm + 50 mm)/2 = 70 mm

Therefore, the mean gasket length *L*, from (3), is:

*L* = 70 mm ∙π = 219.9 mm = 0.22 m

The leakage rate values calculated from (4) are presented in Table 7.

It can be seen that contact pressure is a significant factor with regard to determining leakage. When the leakage rate is considered with regard to contact pressure, a correlation can be noticed. The leakage of the pure fuels and biofuels through the AFO gasket was smaller than through the AFM gasket at similar final contact pressure values. In the case of the Avgas, it is noticeable that the bio-agent additive increased the leakage with regard to the AFO gasket. The opposite situation was observed in the case of the AFM gasket. In the case of the Jet A-1, the bio-agent additive increased the leakage with regard to the AFO gasket. The opposite situation was observed in the case of the AFM gasket. This indicates that the effect of the bio-additive on the gasket material is inconsistent. The fuel loss for both tested types of gasket materials was at most 1 g after 1000 h of testing, which is two times more than the permissible fuel loss according to DIN 28090-3:2014-11 [12].

## 4. Conclusions

- The conducted research shows that there is an influence of the applied bio-additive on the sealing material; however, the effect is not clear.

The observations resulting from the presented research can be summarized in the following points:

- Bio-Jet A1 deteriorates the contact pressure for both AFO and AFM materials,
- Bio-Avgas deteriorates the contact pressure for the AFO material, but improves it for the AFM material,
- Pure Jet A-1 can be used with both AFO and AFM gaskets, while the pure Avgas can only be used with the AFO gasket,
- Bioadditives in Jet A-1 shift the time *t*_0_ to larger values (approx. 350 h),
- Bioadditives in Avgas have a significant influence on time *t*_0_,
- Bio-Jet A-1 should not be used with AFO or AFM gaskets,
- Bio-Avgas could be used with both AFO and AFM gaskets,
- Bio-additive to Jet A1 and to Avgas deteriorates tightness in the case of the AFO material,
- Bio-additive to Jet A-1 and to Avgas increases tightness in the case of the AFM material,
- An increase in leakage in the case of the AFO gasket and the bio-agent coincides with a decrease in contact pressure in the flange joints.

Further research seems to be advisable, with the question of what causes the excessive drop in contact pressure after 1000 h in the case of some fuel and gasket material configurations needing to be answered. Moreover, why in some cases does this drop cause increased leakage, and in some cases not? It seems justified to continue research in the direction of changes in the internal structure of the composite, which is a gasket, under the influence of bio-additives. This will help to find out why in some cases the performance of the gasket deteriorates and in some it does not.

In addition, the biofuel environment did not affect the physical condition of the gasket. The visual inspection proved that a long lasting contact of the sealing material with the biofuel environment did not lead to failure or any abnormalities.

It is worth mentioning that the content of bio-additives in the tested fuels was not high, and the possibility of supplying aircraft engines with fuels with a concentration of up to 50% means that it is advisable to continue the research. Further studies are planned with the use of aviation fuels with a higher concentration of bio-additives, it is planned to investigate the effect of other bio-additives on sealing materials, as well as to test other types of materials used for gaskets of flange joints.

## Figures and Tables

**Table 1:** Experimental contact pressure values of the AFO gasket for different fuels.

Interval Time, h	Bio-Avgas, MPa	Bio-Jet A-1, MPa	Avgas, MPa	Jet A-1, MPa
0	30.0	30.0	30.0	30.0
24	18.5	17.8	18.2	17.4
100	15.4	14.3	15.0	14.7
200	14.2	13.3	13.8	13.9
500	13.0	10.2	12.1	12.9
1000	10.1	6.7	11.6	12.5

**Table 2:** Experimental contact pressure values of the AFM gasket for different fuels.

Interval Time, h	Bio-Avgas, MPa	Bio-Jet A-1, MPa	Avgas, MPa	Jet A-1, MPa
0	30.0	30.0	30.0	30.0
24	19.5	16.6	16.4	18.7
100	18.0	14.7	13.9	15.4
200	16.4	13.5	12.7	13.7
500	13.7	11.0	10.8	13.1
1000	10.8	8.5	6.3	11.3

**Table 3:** Experimental contact pressure drop values of the AFM and AFO gaskets for different fuels at the 500–1000 h time interval.

Interval Time 500–1000 h	Bio-Avgas, MPa	Bio-Jet A-1, MPa	Avgas, MPa	Jet A-1, MPa
AFO	2.9	3.5	0.5	0.4
AFM	2.9	2.5	4.5	1.8

**Table 4:** The coefficients {*A*, *B*, *C*} fitted to the experimental data using the least square approximation method.

	Coefficients	Bio-Avgas	Bio-Jet A-1	Avgas	Jet A-1
AFO	*A*	−0.0588	−0.0505	−0.0559	−0.0585
	*B*	−0.845	−1.32	−0.982	−0.639
	*C*	30.0	30.0	30.0	30.0
AFM	*A*	−0.0614	−0.0526	−0.0521	−0.0592
	*B*	−1.82	−1.13	−0.887	−0.691
	*C*	30.0	30.0	30.0	30.0

**Table 5:** Fuels weight reduction during the tests (in grams) for the AFO gasket.

Interval Time, h	Bio-Avgas, g	Bio-Jet A-1, g	Avgas, g	Jet A-1, g
24	0.012	0.009	0.010	0.0082
100	0.070	0.043	0.051	0.030
200	0.13	0.082	0.10	0.074
500	0.29	0.21	0.25	0.17
1000	0.58	0.41	0.50	0.30

**Table 6:** Fuels weight reduction during the tests (in grams) for the AFM gasket.

Interval Time, h	Bio-Avgas, g	Bio-Jet A-1, g	Avgas, g	Jet A-1, g
24	0.013	0.015	0.020	0.024
100	0.061	0.062	0.093	0.10
200	0.14	0.16	0.18	0.21
500	0.34	0.33	0.45	0.50
1000	0.64	0.68	0.90	1.00

**Table 7:** Leakage rate of fuel *λ*, mg/(m∙s).

Fuels	AFO, mg/(m∙s)	AFM, mg/(m∙s)
Bio-Avgas	7.3 × 10^−4^	8.1 × 10^−4^
Bio-Jet A-1	5.1 × 10^−4^	8.6 × 10^−4^
Avgas	6.3 × 10^−4^	11.4 × 10^−4^
Jet A-1	3.8 × 10^−4^	12.6 × 10^−4^

## Data Availability

Not applicable.
